# Antioxidant and Anti-Inflammatory Effects of White Mulberry (*Morus alba* L.) Fruits on Lipopolysaccharide-Stimulated RAW 264.7 Macrophages

**DOI:** 10.3390/molecules26040920

**Published:** 2021-02-09

**Authors:** Jae Sik Yu, Sung Ho Lim, Seoung Rak Lee, Chang-Ik Choi, Ki Hyun Kim

**Affiliations:** 1 School of Pharmacy, Sungkyunkwan University, Suwon 16419, Korea; jsyu@bu.edu (J.S.Y.); davidseoungrak@gmail.com (S.R.L.); 2 BK21 FOUR Team and Integrated Research Institute for Drug Development, College of Pharmacy, Dongguk University-Seoul, Goyang 10326, Korea; 93sho617@naver.com; 3 Integrated Research Institute for Drug Development, College of Pharmacy, Dongguk University-Seoul, Goyang 10326, Korea

**Keywords:** *Morus alba* L., white mulberry fruits, antioxidant activity, anti-inflammatory activity, RAW 264.7 macrophages, UHPLC-QTOF-HRMS

## Abstract

In this study, the protective effects of white mulberry (*Morus alba*) fruits on lipopolysaccharide (LPS)-stimulated RAW 264.7 macrophages were investigated. The ethanol (EtOH) extract of white mulberry fruits and its derived fractions contained adequate total phenolic and flavonoid contents, with good in vitro antioxidant radical scavenging activity. The extract and fractions also markedly inhibited ROS generation and antioxidant activity. After treatment with the EtOH extract and its fractions, LPS stimulation-induced elevated nitric oxide (NO) production was restored, which was primarily mediated by downregulation of inducible NO synthase expression. A total of 20 chemical constituents including flavonoids, steroids, and phenolics were identified in the fractions using ultra-high-performance liquid chromatography (UHPLC)-quadrupole time-of-flight (QTOF) high-resolution mass spectrometry (HRMS). These findings provide experimental evidence of the protective effects of white mulberry fruit extract against oxidative stress and inflammatory responses, suggesting their nutraceutical and pharmaceutical potential as natural antioxidant and anti-inflammatory agents.

## 1. Introduction

Inflammation, a major mechanism mediating innate and adaptive immunity, is a complex physiological response that protects the organism against foreign harmful stimuli such as pathogens, particles, and viruses. Inflammation is primarily classified as acute and chronic based on the underlying mechanisms and processes. Cellular and molecular processes of chronic inflammation are varied and depend on the organ involved and, thus, are closely associated with the development and deterioration of many chronic diseases including cardiovascular, neurological, pulmonary, metabolic, endocrine, and autoimmune disorders as well as cancer [1]. Following the initiation of inflammatory responses, immune system cells release pro-inflammatory cytokines such as tumor necrosis factor (TNF)-α, interleukin (IL)-1β, and IL-6, which induce the generation of reactive oxygen species (ROS) [2]. Persistent inflammation can cause cellular injury or hyperplasia following ROS overproduction by inflammatory cells. In addition, cellular antioxidant systems activate genes involved in DNA repair in response to ROS-induced DNA damage [3]. Similarly, excessive oxidative stress increases the levels of inflammatory cytokines and related molecules [4].

Macrophages have been found to play a key role in the host defense system, where they are involved in many immunologic functions including inflammatory modulation and removal of apoptotic cells. Macrophages are activated by exogenous mediators such as lipopolysaccharide (LPS), an endotoxin expressed in the cell walls of gram-negative bacteria. This phenomenon is considered the first step in the inflammatory process, and many studies of protective effects mediated by anti-inflammatory, immune-modulating, and antioxidant activities have been performed using LPS-treated macrophage cells [5,6].

The white mulberry tree (*Morus alba* L.), a perennial plant belonging to the Moraceae family, is used in traditional medicine and widely known as an important food source for the silkworm [7]. Additionally, the mulberry tree has economic and ecological importance as it is known for its rapid growth and biomass production [8]. Its fruit is a multiple fruit with a sweet flavor, and it is extensively consumed in various forms including as tea, dessert, and beverages worldwide [7]. Specifically, the mulberry fruit, which is rich in beneficial nutrients, contain secondary metabolites that have pharmacological activities such as antidiabetic, antioxidant, anti-obesity, and anti-inflammatory effects [9].

Previous phytochemical studies of the mulberry fruit have identified secondary metabolites and phytochemicals including flavonoids, anthocyanins, carotenoids, triterpenoids, and phenols, serving as good sources of substances that mediate the various therapeutic effects mentioned. In the present study, we investigated the protective effects of white mulberry fruits on LPS-stimulated RAW 264.7 macrophages by determining cell viability and antioxidant and anti-inflammatory activities. Additionally, the extracts of white mulberry fruits were comprehensively analyzed to identify the active chemical constituents using ultra-high performance liquid chromatography (UHPLC)-quadrupole time-of-flight (QTOF)-high-resolution mass spectrometry (HRMS).

## 2. Results

### 2.1. Total Phenolic and Flavonoid Contents

The results showed that the ethanol (EtOH) extract of the white mulberry fruits and its derived fractions contained adequate total phenolic (from 102.0 to 204.3 mg garlic acid equivalent (GAE)/g) and flavonoid (from 55.1 to 74.9 mg catechin equivalent (CAE)/g) contents. Furthermore, the highest total phenolic content was found in the *n*-butanol (BuOH) fraction (204.3 ± 4.7 mg GAE/g), the highest flavonoid content was in the ethyl acetate (EA) fraction (74.9 ± 4.7 mg CAE/g), while the hexane (HX) fraction exhibited the lowest values of both contents (Table 1).

### 2.2. Cell Viability

**Figure 1:**
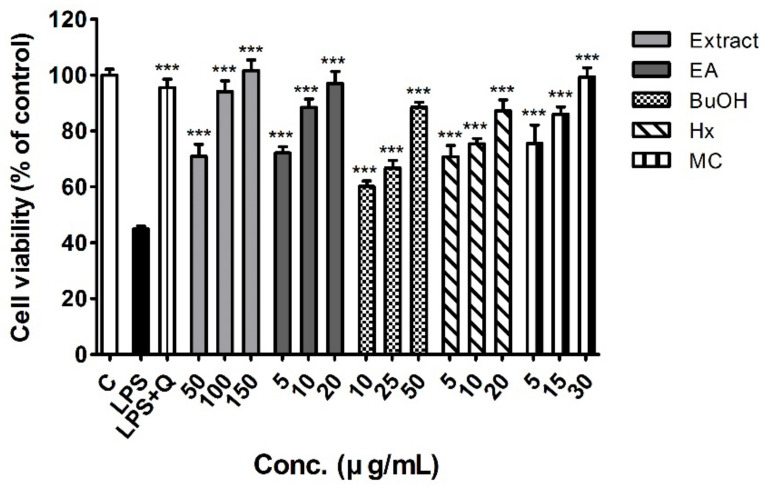
Effects of the EtOH extract and fractions of white mulberry fruits on cellular viability of RAW 264.7 macrophages stimulated with lipopolysaccharide (LPS, 2 μg/mL). Data are expressed as means ± SD (*n* = 5). *** *p* < 0.001 vs. LPS treatment.

Figure 1 shows the viability of RAW 264.7 cells treated with different concentrations of the EtOH extract and fractions of white mulberry fruits, which had no significant effect on cellular viability. There was an approximately 55% reduction in cell viability after pre-incubation of RAW 264.7 cells with LPS 2 μg/mL, which was completely restored by treatment with 10 μM quercetin used as a positive control. In addition, LPS-stimulated RAW 264.7 cells co-treated with white mulberry fruit extract and fractions showed dose-dependent enhancement of viability (all *p* < 0.001, Figure 1). The differences among the fractions were evaluated with multiple comparison analysis at the lowest concentration tested (5 μg/mL for EA, HX and MC fractions; 10 μg/mL for BuOH fraction). The EA, HX and MC fractions exhibited better cell viability compared to BuOH fraction (*p* < 0.01).

### 2.3. Antioxidant Activity

Comparative results of the in vitro antioxidant assays are presented in Table 1. The EA, BuOH, and MC fractions of the EtOH extract showed good 2,2-diphenyl-1-picrylhydrazyl (DPPH) radical scavenging activities, and the EA fraction exhibited the most potent activity (half-maximal inhibitory concentration (IC_50_), 133.6 ± 4.7 μg/mL). The EtOH crude extract and HX fraction did not exhibit DPPH radical scavenging activity at the concentration range tested (IC_50_ > 1000 μg/mL).

The extract and all fractions of white mulberry fruits except for the HX fraction, exhibited 2,2′-azino-bis(3-ethylbenzothiazoline-6-sulfonic acid) (ABTS) radical scavenging activities at the concentration range tested. The EA and MC fractions showed higher activity (IC_50_, 216.6 ± 28.8 and 218.1 ± 22.6 μg/mL, respectively) than the extract and other fractions. The ferric reducing antioxidant power (FRAP) values of the studied samples ranged from 0.505 to 3.727 mmol Fe^2+^/g. Similar to the results of the DPPH and ABTS assay, those of the FRAP assay showed that the EA fraction (3.727 ± 0.055 mmol Fe^2+^/g) exhibited the highest value of all tested samples.

**Figure 2:**
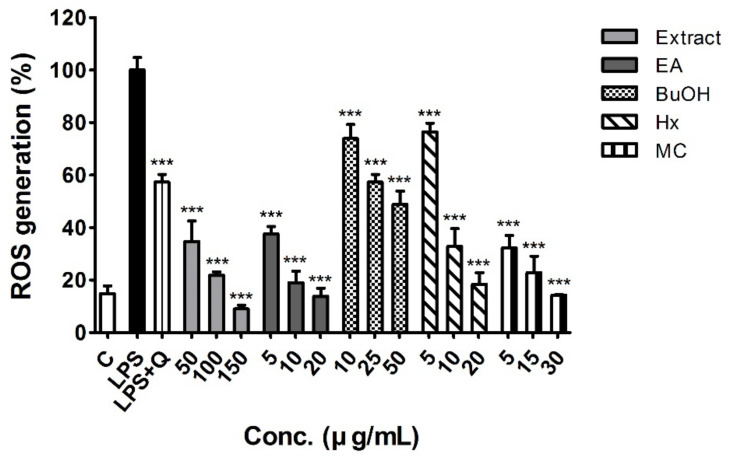
Effects of the EtOH extract and fractions of white mulberry fruits on reactive oxygen species (ROS) generation in LPS-stimulated RAW 264.7 macrophages. Data are expressed as means ± SD (n = 5). *** *p* < 0.001 vs. LPS treatment.

Intracellular ROS levels of LPS-stimulated RAW 264.7 macrophages were 5-fold higher than those of the control, whereas co-treatment with white mulberry fruit extract and fractions significantly inhibited LPS-induced ROS generation. Even at the lowest concentration, the EtOH extract and EA and MC fractions inhibited ROS production (34.8%, 37.6%, and 21.2% of LPS treatment levels, respectively) more than the positive control (quercetin 10 μM, 57.4% of LPS treatment levels) (Figure 2). After the multiple comparison analysis, EA and MC fractions showed significantly lower ROS generation than that in BuOH and HX fractions, respectively (*p* < 0.001).

The antioxidant activities of superoxide dismutase (SOD), catalase (CAT), and glutathione peroxidase (GPx) were also significantly enhanced by treatment with white mulberry fruits extract and fractions at the lowest studied concentrations, as described in Table 2. Significant differences between the fractions were observed only in the GPx activity; EA fraction exhibited better GPx activity than HX fraction (*p* < 0.01), and MC fraction had the highest GPx activity (2.700 ± 0.044 nmol/min), reaching the significant levels compared to BuOH (*p* < 0.05) and HX (*p* <0.01) fraction.

### 2.4. Anti-Inflammatory Activity

**Figure 3:**
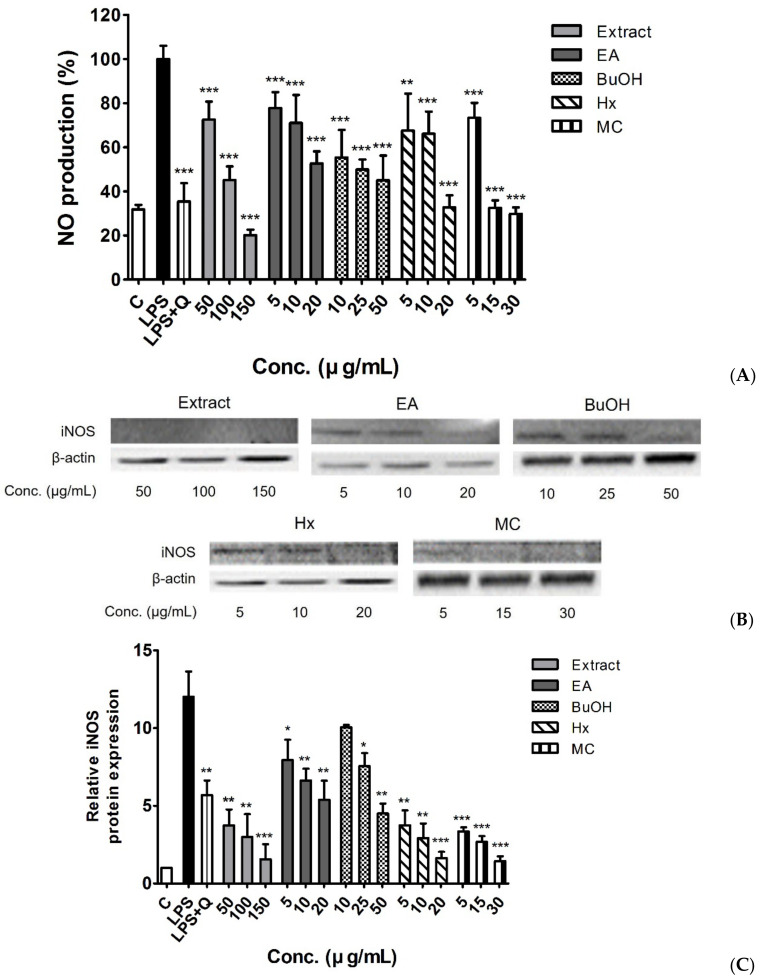
Effects of the EtOH extract and fractions of white mulberry fruits on NO production (**A**) and iNOS protein expression (**B**,**C**) in LPS-stimulated RAW 264.7 macrophages. Data are expressed as means ± SD (*n* = 3). * *p* < 0.05, ** *p* < 0.01, and *** *p* < 0.001 vs. LPS treatment.

As shown in Figure 3A, nitric oxide (NO) levels were highly increased by LPS treatment of RAW 264.7 macrophages. The LPS-induced increase in NO production was significantly inhibited by quercetin 10 μM (35.4% of LPS treatment levels) as well as the white mulberry fruit extract and fractions in a concentration-dependent manner. Interestingly, the EtOH extract and MC and HX fractions exhibited potent inhibitory effects on NO production at the maximum tested concentrations (20.2%, 29.9%, and 32.8% of LPS treatment levels, respectively), which was slightly different from the results of the antioxidant activity analysis. In multiple comparison analysis, the significant differences were observed only between EA and BuOH fractions (*p* < 0.05).

Figure 3B,C show the relative protein expression levels of inducible NO synthase (iNOS) in LPS-treated RAW 264.7 macrophages. Treatment with the white mulberry fruit extract and fractions resulted in lower iNOS levels than that observed with LPS-treatment only. Notably, the EtOH extract and HX and MC fractions showed a higher suppression of iNOS expression than the positive control treatment did at all studied concentration ranges. These findings were consistent with the results of multiple comparison analysis; both HX and MC fractions showed significantly lower iNOS expression than that in EA and BuOH fractions (*p* < 0.01 for EA vs. HX, *p* < 0.001 for others).

### 2.5. Mass Spectral Identification and Qualitative Analysis of Extracts

QTOF-MS is a widely used tool in the field of metabolomics that yields high mass accuracy and elucidates the elemental composition of compounds [10]. HRMS data yield valuable information that enables screening of the masses of secondary metabolites and, thus, are another powerful tool because compounds can be identified without using actual reference standards. The chemical constituents of the *M. alba* fruit extracts were qualitatively analyzed using UHPLC-QTOF-HRMS. In total, 20 chemical constituents were detected in the fractions of the crude extract (Table 3), and all the metabolites were characterized based on the MS data, which was interpreted based on currently available literature [11,12,13,14,15,16]. The exact mass of the reported compounds was compared with the obtained mass data to generate the parts per million (ppm) value. A parts per million value between the theoretical and measured exact mass of approximately ±50 ppm indicated the compound was a positive match [17]. Thus, lower parts per million values indicate a higher probability of the measure compound existing in the extract. The exact masses were calculated based on the possible proton and sodium adducts under positive ionization. The structures of these compound have been identified and characterized from *M. alba* in previously reported studies [11,12,13,14,15,16].

## 3. Discussion

The in vitro antioxidant assays performed in this study (DPPH, ABTS, and FRAP assays) are simple and most commonly used in the early screening of antioxidant properties of vegetable and fruit extracts and products [18]. DPPH is a highly colored and stable free radical, which in the presence of antioxidant substances is reduced to the non-radical 2,2-diphenyl-1-picrylhydrazine, with a loss of its violet color [19]. The ABTS assay measures the capacity of an antioxidant to scavenge ABTS radicals (ABTS^+^) generated by reacting the parent compound with a strong oxidizing agent such as potassium persulfate. It can be used over a wide pH range in both aqueous and organic solvent systems [20]. The FRAP assay directly evaluates total antioxidant power, where the ferric-tripyridyltriazine (Fe^3+^-TPTZ) complex is reduced to the ferrous form (Fe^2+^) at low pH, with an intense blue color [21]. In this study, the EtOH extract and fractions of white mulberry fruits exhibited appropriate DPPH and ABTS radical scavenging effects and FRAP values, which were especially high with the EA and MC fractions. On the contrary, the HX fraction showed the lowest antioxidant activity, with the IC_50_ values for DPPH and ABTS assays over the upper concentration limit tested in this study (IC_50_ > 1000 μg/mL) (Table 1). These findings are very similar to the results of other previously published study [22]. The results suggest that the protective effect of white mulberry fruits against oxidative stress is primarily mediated by constituents of the EA and MC fractions.

Intracellular levels of free radicals can damage various cell constituents and activate specific signaling pathways, which both affect numerous cellular process linked to aging and the development of related diseases [23]. Overproduction of ROS and reduced antioxidant capacity can result in a redox imbalance, inducing the inflammatory response and oxidative stress eventually leading to the formation of various pathophysiological lesions [24]. Consistent with the results of previous studies [25,26,27], in this study, LPS increased ROS generation in stimulated RAW 264.7 macrophages (Figure 2). Co-treatment of LPS-stimulated RAW 264.7 macrophages with white mulberry fruit extract and fractions resulted in significant reductions in ROS levels. At the maximum concentrations used in this assay, all studied samples, except for the BuOH fraction, showed similar inhibition of ROS production to that in the control, which was not stimulated by LPS. Furthermore, the EA and MC fractions induced lower ROS levels than the positive control (quercetin 10 μM) at the lowest tested concentration, indicating the results were similar to those of the in vitro antioxidant assays (Figure 2).

Antioxidant enzymes such as SOD, GPx, and CAT stabilize or inactivate the detrimental effects of free radicals on cellular components. They also inhibit the oxidizing chain reaction to minimize free radical-induced cellular and molecular damages. By reducing cellular exposure to free radicals, antioxidant enzymes contribute to decreasing the risk for various associated health problems including the physiological manifestations of aging, cardiovascular diseases, diabetes, neurodegenerative diseases, and cancer [28]. SOD establishes the first-line defense system against superoxide radicals (O^2−^) by catalyzing their breakdown to oxygen and H_2_O_2_ [29]. This ROS-scavenging process of SOD is only effective with the cooperative actions of GPx and CAT, during which H_2_O_2_ undergoes further degradation [30]. We found that the EtOH extract and all fractions of white mulberry fruits at the lowest concentrations used in this study simultaneously and significantly enhanced SOD, GPx, and CAT enzyme capacities, which were suppressed by LPS treatment (Table 2).

NO is a free radical widely distributed in the body that regulates various biological functions including vasodilation, smooth muscle contraction, neuronal signaling, platelet aggregation inhibition, immunological regulation, and inflammatory responses [31,32]. LPS-induced activation of macrophages leads to iNOS expression, resulting in increased NO production [33]. Excessive NO levels have been implicated in cell death, inflammatory responses, and the pathogenesis of several disease states [34,35]. In this study, white mulberry fruit extract and fractions significantly inhibited the production of nitrites in LPS-stimulated macrophage cells, which protected cell viability (Figure 1 and Figure 3A). We also confirmed that protein expression level of iNOS was lower after treatment with white mulberry fruit samples than that before treatment (Figure 3B,C). This observation indicates that the reduction of NO levels in LPS-stimulated RAW 264.7 macrophages treated with white mulberry fruit extract was primarily mediated by downregulation of iNOS expression. To the best of our knowledge, this is the first study to investigate the anti-inflammatory activity and its underlying mechanism of the fractions of *M. alba* fruit.

Table 3 shows the various chemical constituents we identified from the white mulberry fruit fractions using UHPLC-QTOF-HRMS analysis. From the multiple comparison analyses, superior antioxidant and anti-inflammatory activities in EA and MC fractions were confirmed. Most compounds from the EA fraction, which has the highest total flavonoid content (Table 1), were flavonoids or their derivatives. The constituents identified in this study (quercetin, kaempferol, luteolin, astragalin, and taxifolin) have shown various biological health-promoting effects including antioxidant and anti-inflammatory activities mediated through different molecular mechanisms [36,37,38,39,40]. On the other hand, constituents with various chemical structures were found in the MC fraction. Indole is an aromatic heterocyclic compound commonly distributed in nature. Many well-known indole derivatives have been developed as pharmaceutical agents such as nonsteroidal anti-inflammatory drugs (indomethacin and etodolac), antimigraine agents (sumatriptan and naratriptan), and a non-selective β-blocker (pindolol). In addition, numerous biological activities including antioxidant, anti-inflammatory, analgesic, antimicrobial, antidiabetic, antidepressant, and anticancer have been reported for compounds with an indole nucleus [41]. Loliolide is a monoterpenoid active ingredient found in green algae that exhibits antioxidant, antiviral, anti-inflammatory, anticancer, antimelanogenic, and antiapoptotic properties [42,43,44,45,46]. Odisolane was recently isolated as a novel oxolane derivative from 70% aqueous methanol extracts of *M. alba* fruits [47]. Odisolane significantly inhibited angiogenesis in human umbilical vein vascular endothelial cells, a pathological process that is closely related to chronic inflammation and oxidative stress [12,48]. It was not possible to determine the specific individual effects of the identified compounds because other minor components were also present in the extract and fractions; however, the antioxidant and anti-inflammatory activities of white mulberry fruit could be partially attributed to the complex effects of these active constituents.

The limitation of this study is that the signaling pathway associated with anti-inflammatory effects of white mulberry fruits were not fully identified. Nuclear factor (NF)-κB induces pro-inflammatory cytokines, chemokines, and adhesion molecules that are essential for both innate and adaptive immune responses [49]. NF-κB has been known to play a role in the expression of iNOS and another well-known inflammatory marker, COX-2. Consequently, we also evaluated the effects of white mulberry fruit extract and fractions on the NF-κB signaling pathway and COX-2 expression. However, there was no significant change in NF-κB p65 or COX-2 protein expression following treatment with white mulberry fruit extract and fractions (data not shown), although they are overexpressed in RAW 264.7 macrophages after LPS stimulation. Several studies have reported that the expression of iNOS and COX-2 is also affected by mitogen-activated protein kinase (MAPK), which is involved in the regulation of cell growth, differentiation, and apoptosis [47,50,51,52]. Therefore, further investigations are needed to determine the possible inactivation of the MAPK pathway by white mulberry fruit.

## 4. Materials and Methods

### 4.1. Materials

(±)-Catechin, copper (II) chloride, 2′,7′-dichlorofluorescin diacetate (DCF-DA), dimethyl sulfoxide (DMSO), ethylenediaminetetraacetic acid (EDTA), Folin-Ciocalteu’s phenol reagent, gallic acid, glutathione (GSH), GSH reductase, hydrogen peroxide, 3-(4,5-dimethylthiazol-2-yl)-2,5-diphenyltetrazolium bromide (MTT), LPS, nicotinamide adenine dinucleotide phosphate (NADPH), nitroblue tetrazolium (NBT), phosphate-buffered saline (PBS), quercetin, xanthine, and xanthine oxidase were purchased from Sigma-Aldrich (St. Louis, MO, USA).

The Griess reagent system kit was purchased from Promega (Madison, WI, USA). Dulbecco’s modified Eagle’s medium (DMEM), fetal bovine serum (FBS), and penicillin/streptomycin were purchased from Thermo Fisher Scientific (Waltham, MA, USA). Primary antibodies for iNOS and β-actin were purchased from Cell Signaling Technology (Danvers, MA, USA). Goat anti-rabbit IgG secondary antibody and other chemical reagents were purchased from Merck Millipore (Burlington, MA, USA).

### 4.2. Plant Material, Extraction, and Preparation of Fractions

Mulberry fruits (*M. alba*) were acquired from the Kyungdong Market (Woori Herb), Seoul, Korea, in January 2014. The material was verified by one of the authors (K.H.K.), and a voucher specimen (MA 1414) was deposited in the herbarium of the School of Pharmacy, Sungkyunkwan University, Suwon, Korea. The *M. alba* fruits (0.9 kg) were dried in a hot air oven at 60 °C and the dried materials were extracted with 70% aqueous EtOH and filtered using Whatman filter paper No. 42 three times at room temperature. The filtrate was evaporated in vacuo to obtain the crude EtOH extract (140 g). The extract was dissolved in deionized water and then solvent-partitioned with 800 mL each of HX, MC, EA, and BuOH three times, yielding 2.8 8.5, 3.3, and 13.9 g of the fractions, respectively. Concentrated extracts and fractions were subsequently lyophilized and stored at −20 °C prior to analysis.

### 4.3. Determination of Total Phenolic Content

The total phenolic content was determined using the Folin-Ciocalteu method with some modifications [53]. Each sample (100 μL) was mixed with 200 μL Folin-Ciocalteu reagent and allowed to react for 1 min. Following the addition of 3 mL 5% sodium carbonate (Na_2_CO_3_), the mixtures were incubated for 60 min at room temperature in the dark. The absorbance was measured at a wavelength of 725 nm using a microplate spectrophotometer (xMark, Bio-Rad, Hercules, CA, USA). Gallic acid was used as the standard, and the total phenolic content were determined from calibration curves for gallic acid (*y* = 2.4652*x* + 0.008, *r*^2^ = 0.9998). Results were expressed as milligrams of gallic acid equivalents per gram of sample (mg GAE/g).

### 4.4. Determination of Flavonoid Content

The flavonoid content was determined using the aluminum chloride (AlCl_3_) method with some modifications [54]. Each sample (100 μL) was mixed with 150 μL sodium nitrite (NaNO_2_) and allowed to react for 5 min. Then, 300 μL 10% AlCl_3_ solution and 1 mL 1 M sodium hydroxide were added, and the absorbance was measured at a wavelength of 510 nm using a microplate spectrophotometer at 510 nm. (±)-Catechin was used as the standard, and the flavonoid content were determined from calibration curves for (±)-catechin (*y* = 0.9472*x* + 0.0011, *r*^2^ = 0.9964). Results were expressed as milligrams of catechin equivalents per gram of sample (mg CAE/g).

### 4.5. Cell Culture

Murine RAW 264.7 macrophage cells were cultured in DMEM containing 4 mM L-glutamine, 4.5 g/L glucose, and sodium pyruvate supplemented with 10% FBS and 1% penicillin/streptomycin. Cells were maintained in a humidified atmosphere with 5% carbon dioxide (CO_2_) at 37 °C.

### 4.6. Cell Viability

Cell viability was assessed using the MTT assay with some modifications [55,56,57,58,59]. Briefly, cell were seeded in 96-well plates (2 × 10^4^ cell/well) and treated with 0.5% DMSO (control), 10 μM quercetin (positive control), or different concentrations of the crude extract of white mulberry fruit (50–150 μg/mL) and the EA (5–20 μg/mL), BuOH (10–50 μg/mL), HX, (5–20 μg/mL), and MC (5–30 μg/mL) fractions in the absence or presence of LPS (2 μg/mL in PBS) for 24 h. Following treatment, 20 μL MTT solution (5 mg/mL in PBS) was added to each well, and the plates were incubated at 37 °C in an atmosphere of 5% CO_2_ for 2 h. Then, the supernatant was removed, and 100 μL DMSO was added to each well to dissolve any formazan crystals that developed. The absorbance of each well was measured at 570 nm using a microplate spectrophotometer.

### 4.7. Antioxidant Activity

#### 4.7.1. DPPH Radical Scavenging Assay

The DPPH radical scavenging activity was determined using the method of Blois [60] with some modifications. Briefly, 50 μL 0.2 mM DPPH solution was added to the same volume of each sample at a concentration range of 10–1000 μg/mL and incubated at room temperature for 15 min in the dark. The absorbance was measured using a microplate spectrophotometer at 517 nm. The scavenging activity was calculated as follows: DPPH scavenging activity (%) = ((A_0_ − A_c_)/A_0_) × 100 (where, A_0_ and A_c_ are the absorbance of the control and sample, respectively).

#### 4.7.2. ABTS Radical Scavenging Assay

The ABTS radical scavenging activity was determined using the method of Arts et al. [61] using ABTS with some modifications. Briefly, 7 mM ABTS and 2.45 mM potassium persulfate were mixed (1:1) and incubated for at room temperature 24 h in the dark. The ABTS solution was diluted in 100% methanol to obtain an absorbance of 0.70 ± 0.02 at 734 nm. Then, 50 μL diluted ABTS solution was added to the same volume of each sample at a concentration range of 10–1000 μg/mL and incubated for at room temperature 5 min in the dark. The absorbance was measured using a microplate spectrophotometer at 734 nm. The scavenging activity was calculated as follows: ABTS scavenging activity (%) = ((A_0_ − A_c_)/A_0_) × 100 (where A_0_ and A_c_ are the absorbance of the control and sample, respectively).

#### 4.7.3. FRAP Assay

The FRAP assay was performed using the method of Benzie and Strain [21] with some modifications. The FRAP reagent was prepared by mixing 300 mM acetate buffer (pH 3.6), 10 mM 2,4,6-tris(2-pyridyl)-s-triazine, and 20 mM ferric chloride (FeCl_3_∙6H_2_O) at a 10:1:1 ratio. Then, 175 μL FRAP reagent was added to 25 μL of each test sample at a concentration of 1000 μg/mL and incubated at 37 °C for 4 min. Ferrous sulfate (FeSO_4_∙7H_2_O) was used as the standard, and the absorbance was measured at a wavelength of 593 nm using a microplate spectrophotometer. The results are expressed as millimoles (mmol) of FeSO_4_∙7H_2_O equivalents per gram of sample (mmol Fe^2+^/g).

#### 4.7.4. Measurement of Intracellular ROS Levels

Intracellular ROS levels were measured using the DCF-DA assay as described by Sittisart and Chitsomboon [62]. Cell were seeded in 96-well plates (2 × 10^4^ cell/well), treated with the positive control or different concentrations of crude extract and fractions of white mulberry fruits for 2 h, and then incubated with LPS for 20 h. Then, the supernatant was discarded, and 20 μM DCF-DA in serum-free DMEM was added, followed by further incubation at 37 °C for 30 min, protected from light. The supernatant was removed and washed with PBS twice, and then 100 μL PBS was added to each well. The fluorescence intensity was detected at excitation and emission wavelengths of 485 and 535 nm, respectively, using a multi-mode microplate reader (SpectraMax M3, Molecular Devices, San Jose, CA, USA).

#### 4.7.5. Antioxidant Enzyme Capacity Assays

The antioxidant enzyme capacity was assayed in accordance with the methods previously described by Lee et al. [30]. Briefly, RAW 264.7 cells were seeded in 24-well plates (2 × 10^5^ cell/well), treated with the positive control or white mulberry fruits extract and fractions for 2 h, and then they were incubated with LPS for 20 h. The culture medium was removed, and the cells were washed twice and then scraped with 1 mL PBS. Cell suspension were centrifuged at 14,000 rpm at 4 °C for 5 min.

For the determination of SOD activity, cell homogenates were prepared by homogenizing cell suspensions with 0.05 M sodium carbonate buffer (pH 10.2). The final reaction mixture consisted of 50 μL cell homogenate and 0.05 M sodium carbonate buffer containing 3 mM xanthine, 0.75 mM NBT, 3 mM EDTA, and 1.5 mg/mL bovine serum bovine albumin (BSA). The reaction was initiated by adding 50 μL xanthine oxidase (0.1 mg/mL) and incubating at room temperature for 30 min, and then it was stopped by adding 6 mM copper (II) chloride and centrifuging at 1500 rpm for 10 min. The absorbance of blue formazan in the supernatant was determined at a wavelength of 560 nm.

The assay mixture for determining GPx activity contained 0.1 M phosphate buffer (pH 7.0), 1 mM EDTA, 1.5 mM NADPH, 1 mM sodium azide, 1 unit of GSH reductase, 10 mM GSH, and 100 μL cell lysates. This mixture was incubated at 37 °C for 10 min, and then hydrogen peroxide (H_2_O_2_) was added to each sample at a final concentration of 1 mM, followed by the measurement of activity at a wavelength of 340 nm.

The assay mixture for CAT activity contained 12 μL 3% H_2_O_2_ and 100 μL cell lysates in 50 mM phosphate buffer (pH 7.0), and samples were incubated at 37 °C for 2 min. The absorbance of the samples was measured for 5 min at a wavelength of 240 nm. The variation in absorbance is proportional to the breakdown of H_2_O_2_.

### 4.8. Anti-Inflammatory Activity

#### 4.8.1. Measurement of Intracellular NO levels

The concentration of nitrite, a stable oxidized product of NO, in the cell culture medium was determined using a Griess reagent system kit (Promega). Cells were seeded in 96-well plates (2 × 10^4^ cell/well) and treated with the positive control or different concentrations of white mulberry fruits crude extract and fractions for 2 h, followed by LPS for 24 h. Then, 50 μL samples of the supernatant from the treated culture medium was mixed with 50 µL 1% sulfanilamide in 5% phosphoric acid and incubated at room temperature for 10 min, protected from light. Then, 50 µL 0.1% *N*-1-napthylethylenediamine dihydrochloride in water was added, followed by incubation at room temperature for 10 min, protected from light. The absorbance was measured at a wavelength of 540 nm using a microplate spectrophotometer. The NO level of each experimental sample was calculated using a NaNO_2_ (0–100 µM) standard curve.

#### 4.8.2. Measurement of iNOS Protein Expression

For Western blot analysis, cells were seeded in 12-well plates (5 × 10^5^ cells/well) and pre-incubated for 2 h with the positive control and different concentrations of the crude extract and each fraction. After LPS stimulation at 37 °C for 20 h in a humidified atmosphere of 5% CO_2,_ cells were washed with PBS, homogenized with lysis buffer containing a protease inhibitor cocktail, and centrifuged at 14,000 rpm (4 °C, 20 min). Each supernatant sample containing an equal total protein amount (20 μg) was loaded for separation using 10% sodium dodecyl sulfate-polyacrylamide gel electrophoresis and then transferred onto a polyvinylidene difluoride membrane.

After blocking with 5% skim milk for 1 h, the membrane was incubated with a primary antibody against iNOS (1:1000 dilution in 5% BSA) at 4 °C overnight, followed by a horseradish peroxidase-conjugated secondary antibody (1:2000 dilution in 5% skim milk) at room temperature for 1 h. The membrane was washed, and immunoreactive bands were detected using the ChemiDoc imaging system with an enhanced chemiluminescence solution kit (Bio-Rad).

### 4.9. Chemical Profiling and Qualitative UHPLC-QTOF-MS Analysis

Extracts of white mulberry fruits were chemically profiled using an Agilent 1290 Infinity II HPLC instrument (Foster City, CA, USA) coupled to a G6545B Q-TOF mass spectrometer (Agilent Technologies). HX, MC, EA, and *n*-BuOH soluble fractions were dissolved in the respective extraction solvents and filtered through 0.45-μm filters before injection. The identified compounds were separated using an Agilent EclipsePlus C18 column (2.1 mm × 50 mm, 1.8 μm; flow rate 0.3 mL/min) maintained at 20 °C. The mobile phrase consisted of 0.1% formic acid in water (solvent A) and 100% acetonitrile (solvent B). The gradient elution was performed on the following schedule: 90% A → 100% B (0–10 min), 100% B (11–16 min), and 90% A (16–20 min) for equilibration before the next injection. The samples were monitored at 210 and 254 nm during the chromatographic run. The mass spectral analysis was performed using the MassHunter software (Agilent, Foster City, CA, USA), and the mass spectrometer conditions were as follows: ionization mode, electrospray ionization (ESI (+)); MS scan range, *m*/*z* 100–1700; nebulizer gas (N_2_) pressure, 35 psi; dry gas (N_2_) flow rate, 8 L/min; drying gas temperature, 225 °C; sheath gas temperature, 320 °C; capillary voltage, 3.5 kV; fragmentor voltage, 100 V; collision energy, 3.0 eV [63]. The exact mass of some of the organic compounds identified from the mass spectral data was compared with theoretical values from previous studies [11,12,13,14,15,16]. The significance of the obtained data was confirmed by the results calculated from the obtained and theoretical data. The accuracy was reported as change (Δ, parts per million (ppm)) and was calculated using the following equation,

$$\frac{\left(mass_{exp}-mass_{calc}\right)}{mass_{exp}}\;\times 10^{6}$$

where, *mass_exp_* and *mass_calc_* are the experimental mass and calculated mass from previously published molecular formulas, respectively [17].

### 4.10. Statistical Analysis

All experiments were replicated three or five times, and assay results are expressed as the means ± SD. The IC_50_ value of the DPPH and ABTS radical scavenging assays was defined as the concentration of sample scavenging 50% of the free radicals. The significance of the difference in mean values between each mulberry fruit sample and control or LPS treatment sample were analyzed using the Student’s *t*-test, and the difference in mean values among mulberry fruit fractions at the lowest concentration (5 μg/mL for EA, HX and MC fractions; 10 μg/mL for BuOH fraction) were analyzed using the one-way analysis of variance (ANOVA) with post-hoc Tukey multiple comparison test. *p*-values < 0.05 were considered statistically significant.

## 5. Conclusions

In conclusion, we demonstrated that white mulberry fruits contain adequate amounts of total phenolics and flavonoids and exhibited beneficial antioxidant and anti-inflammatory properties without cytotoxicity. The present study also reported the chemical constituents of white mulberry fruit extracts, including some associated with observed biological activities of antioxidant and anti-inflammatory agents. Our findings indicate that white mulberry fruits have protective effects against oxidative stress and inflammatory responses, suggesting their nutraceutical and pharmaceutical potential to be developed as natural antioxidant and anti-inflammatory agents.

## Figures and Tables

**Table 1:** Total phenolic and flavonoid contents and antioxidant activities of EtOH extract and fractions of white mulberry fruits ^a^.

	Total Phenolic (mg GAE/g)	Flavonoid (mg CAE/g)	DPPH (IC_50_, μg/mL)	ABTS (IC_50_, μg/mL)	FRAP (mmol Fe^2+^/g)
EtOH extract	173.1 ± 3.9	68.5 ± 8.2	> 1000	890.6 ± 56.6	0.770 ± 0.019
EA fraction	177.9 ± 4.7	74.9 ± 8.6	133.6 ± 4.7	216.6 ± 28.8	3.727 ± 0.055
BuOH fraction	204.3 ± 4.7	70.6 ± 6.9	471.1 ± 35.3	793.2 ± 8.4	1.259 ± 0.026
HX fraction	102.0 ± 1.5	55.1 ± 9.6	> 1000	> 1000	0.505 ± 0.017
MC fraction	124.7 ± 0.6	64.3 ± 2.8	475.0 ± 18.4	218.1 ± 22.6	1.649 ± 0.023

^a^ Data are means ± standard deviation (SD, *n* = 3). BuOH, butanol; EA, ethyl acetate; HX, hexane; MC, methylene chloride. DPPH, 2,2-diphenyl-1-picrylhydrazyl; ABTS, 2,2′-azino-bis(3-ethylbenzothiazoline-6-sulfonic acid); FRAP, ferric reducing antioxidant power; GAE, garlic acid equivalent; CAE, catechin equivalent; IC_50_, half-maximal inhibitory concentration.

**Table 2:** Antioxidant enzyme capacities of EtOH extract and fractions of white mulberry fruits in LPS-treated RAW 264.7 macrophages ^a^.

	SOD (% of Control)	GPx (nmol/min)	CAT (unit/mL)
Control (0.5% DMSO)	100.0	2.674 ± 0.029	0.147 ± 0.007
LPS 2 μg/mL	64.6 ± 0.2 ^###^	2.292 ± 0.092 ^##^	0.120 ± 0.007 ^##^
LPS + Quercetin (10 μM)	93.7 ± 5.1 ***	2.547 ± 0.039 **	0.163 ± 0.005 **
LPS + EtOH Extract (50 μg/mL)	78.7 ± 3.2 **	2.776 ± 0.025 ***	0.156 ± 0.011 *
LPS + EA fraction (5 μg/mL)	91.1 ± 5.7 **	2.674 ± 0.039 **	0.142 ± 0.004 *
LPS + BuOH fraction (10 μg/mL)	85.7 ± 5.1 **	2.521 ± 0.044 **	0.157 ± 0.010 **
LPS + HX fraction (5 μg/mL)	85.6 ± 2.0 ***	2.471 ± 0.015 *	0.162 ± 0.011 **
LPS + MC fraction (5 μg/mL)	89.1 ± 1.9 ***	2.700 ± 0.044 **	0.146 ± 0.012 *

^a^ Data are means ± SD (*n* = 3). BuOH, butanol; EA, ethyl acetate; HX, hexane; MC, methylene chloride; SOD, superoxide dismutase; GPx, glutathione peroxidase; CAT, catalase. ^##^ *p* < 0.01 and ^###^ *p* < 0.001 vs. control; * *p* < 0.05, ** *p* < 0.01, and *** *p* < 0.001 vs. LPS treatment.

**Table 3:** Chemical constituents identified in the fractions of white mulberry fruits using ultra-high-performance liquid chromatography-quadrupole-time-of-flight-high-resolution mass spectrometry (UHPLC-QTOF-HRMS).

Fractions	Compound	Formula	Theoretical (*m*/*z*)	Experimental (*m*/*z*)	Adduct	Δ (ppm) ^a^
Hexane	Ar-Turmerone	C_15_H_20_O	217.1592	217.1585	M + H	−3.22
	(2*S*,4a*R*,4b*R*,6a*R*,8*S*,10a*R*,10b*R*,12a*S*)-8-(Acetyloxy)hexadecahydro-2,4a,4b,7,7,10a-hexamethyl-2-(3-oxobutyl)-1(2*H*)-chrysenone	C_30_H_48_O_4_	473.3631	473.3621	M + H	−2.11
	Stigmasterol	C_29_H_48_O	413.3783	413.3779	M + H	−9.68
Methylene chloride	Odisolane	C_8_H_14_O_4_	175.097	175.0979	M + H	5.14
	3-Benzofurancarboxyaldehyde	C_9_H_6_O_2_	147.0446	147.0437	M + H	−6.12
	Loliolide	C_11_H_16_O_3_	197.1172	197.1178	M + H	3.04
	(*R*)-5-Hydroxypyrrolidin-2-one	C_4_H_7_NO_2_	102.0547	102.0555	M + H	7.84
	Methyl *R*-pyroglutamate	C_6_H_9_NO_3_	144.0661	144.0655	M + H	−4.16
	Indole	C_8_H_7_N	118.0657	118.0646	M + H	−9.32
Ethyl acetate	Quercetin	C_15_H_10_O_7_	303.0505	303.0499	M + H	−1.98
	Kaempferol	C_15_H_10_O_6_	287.0556	287.0549	M + H	−2.44
	Luteolin	C_15_H_10_O_6_	287.0556	287.0546	M + H	−3.48
	Astragalin	C_21_H_20_O_11_	449.1084	449.1082	M + H	−4.45
	Taxifolin	C_15_H_12_O_7_	305.0661	305.0656	M + H	−1.64
	Morrole A	C_14_H_21_NO_5_	284.1498	284.1489	M + H	−3.17
	Methyl chlorogenate	C_17_H_20_O_9_	369.1186	369.1181	M + H	−1.35
*n*-Butanol	Quercetin 3-*O*-β-glucoside	C_21_H_20_O_12_	465.1033	465.1028	M + H	−1.08
	Kaempferol 3-*O*-β-rutinoside	C_27_H_30_O_15_	595.1663	595.1659	M + H	−6.72
	Rutin	C_27_H_30_O_16_	611.1612	611.1609	M + H	−4.91
	Butyl L-pyroglutamate	C_9_H_15_NO_3_	186.113	186.1127	M + H	−1.61

^a^ Deviation of measured *m*/*z* from calculated *m*/*z* values for a pseudomolecular ion generated from the molecular formula.

## Sample Availability

Samples of the compounds are not available from the authors.
